# Management of a patient with severe insulin resistance

**DOI:** 10.1210/jcemcr/luag200

**Published:** 2026-07-22

**Authors:** Brandon Arulanandam, Natasha Garfield

**Affiliations:** Department of Medicine, McGill University Faculty of Medicine and Health Sciences, Montreal, QC H3G 2M1, Canada; Department of Medicine, Division of Adult Endocrinology and Metabolism, McGill University Health Centre, Montreal, QC H4A 3J1, Canada

**Keywords:** diabetes, insulin resistance

## Abstract

We report the case of a 48-year-old man with type 2 diabetes with severe insulin resistance, requiring more than 1000 units of insulin per day. Our case highlights the challenges in treating patients with severe insulin resistance, including ruling out secondary causes and using less-commonly prescribed therapies such as U-500 insulin.

## Introduction

Diabetes is a chronic condition affecting 15% of the Canadian population, and the majority of these cases are type 2 diabetes [1]. Type 2 diabetes exists on a spectrum, ranging from predominant insulin resistance to a combination of insulin resistance and impaired insulin secretion [2]. Severe insulin resistance is defined as needing >200 units per day of U-100 insulin [3], which can be challenging for physicians to manage. In such situations, a workup to exclude secondary causes of diabetes is warranted, as managing the underlying condition can assist in improving glycemic control. We report the case of a 48-year-old man with type 2 diabetes with severe insulin resistance, requiring more than 1000 units of insulin per day.

## Case presentation

A 48-year-old man with a 28-year history of type 2 diabetes was referred to our diabetes service for management of severe insulin resistance. He had no known complications of diabetes at the time of initial evaluation. A recent glycated hemoglobin (A1C) was 8.4% (SI: 68 mmol/mol) (reference range, 4.8%-6.0%; [SI: 29-42 mmol/mol]).

His past medical history was significant for hypertension, dyslipidemia, sleep apnea, hepatic steatosis, and adrenal myelolipoma. Family history was significant for type 2 diabetes in his father, uncle, and grandmother.

Earliest available records from 2017 showed an A1C of 7.3% (SI: 56 mmol/mol) while on treatment with 300 units of insulin lispro per day, 100 units of insulin glargine twice daily, metformin 2 g daily, and liraglutide 3 mg daily. In 2018, he achieved an A1C of 6.8% (SI: 51 mmol/mol) and liraglutide was discontinued for undocumented reasons. He maintained an A1C in this range with the same regimen until 2020 when his A1C deteriorated to 8.8% (SI: 73 mmol/mol). He was started on the FreeStyle Libre continuous glucose monitor, and his insulin glargine was changed to degludec 125 units twice daily, resulting in persistent hyperglycemias and an A1C of 8.7% (SI: 72 mmol/mol) in 2021, when dapagliflozin 10 mg was added.

In 2022, his A1C improved back to 8.2% (SI: 66 mmol/mol), but he developed recurrent mycotic genital infections and thus dapagliflozin was stopped while increasing insulin degludec to 300 units daily, with subsequent improvement to 7.2% (SI: 55 mmol/mol). Semaglutide 1 mg weekly was then added, although his A1C worsened to 8.6% (SI: 70 mmol/mol), at which point his diabetes care was transferred to us.

At the time of referral, the patient was on >1000 units of insulin daily (lispro 300-400 units pre-breakfast, 100 units pre-lunch, 300-400 units pre-dinner and 300 units of insulin degludec daily) as well as metformin 1 g twice a day and semaglutide 1 mg weekly.

He was using the FreeStyle Libre 2 continuous glucose monitor with a time-above-range of 82%.

## Diagnostic assessment

Physical examination was notable for a weight of 172 kg, height 203 cm (body mass index [BMI] of 41.7 kg/m^2^), blood pressure of 142/90 mmHg with a deep voice and macrognathia, as well as acanthosis nigricans at the neck and axilla with multiple skin tags, white abdominal striae, lipohypertrophy on his abdomen, generalized skin xerosis, and hyperpigmentation of his shins bilaterally. Waist circumference was not documented in the chart.

After initial consultation with our diabetes clinic, we completed the workup for relevant secondary causes of diabetes as shown in Table 1.

**Table 1:** Workup of secondary causes of diabetes in our patient

Etiology	Diagnostic test	Patient's result [normal range]
Cushing syndrome	1 mg dexamethasone suppression (cortisol)	0.98 μg/dL (SI: 27 nmol/L) (reference range, 3.7-19.4 μg/dL [SI: 102-535 nmol/L]) Suppression if <1.81 μg/dL [SI: 50 nmol/L])
Pheochromocytoma	Norepinephrine urine	50.4 μg 24 hours (SI: 298 nmol/d) (reference range, <74.4 μg 24 hours [SI: <440 nmol/d])
	Epinephrine urine	6.8 μg 24 hours (SI: 37 nmol/d) (reference range, <20.2 μg g/24 hours [SI: <110 nmol/d])
	Dopamine urine	305.3 μg 24 hours (SI: 1993 nmol/d) (reference range, <393.7 μg 24 hours [SI: <2570 nmol/d])
	Normetanephrine urine	26 μg 24 hours (SI: 142 nmol/d) (reference range, <44 μg 24 hours [SI: <240 nmol/d])
	Metanephrine urine	15.2 μg 24 hours (SI: 77 nmol/d) (reference range, <54.2 μg 24 hours [SI: <275 nmol/d])
Acromegaly	IGF-1	138 ng/mL (SI: 138 μg/L) (reference range, 70-200 ng/mL [SI: 70-200 μg/L])
	Growth hormone	0.1 ng/mL (SI: 0.1 μg/L) (reference range, 0-3.8 ng/mL [SI: 0-3.8 μg/L])
Carcinoid	5-HIAA	4.6 mg/24 hours (SI: 24 μmol/d) (reference range, 0.6-7.8 mg/24 hours [SI: 3-41 μmol/d])
	Chromogranin-A	67.3 ng/mL (SI: 67.3 μg/L) (reference range, <82 ng/mL [SI: <82 μg/L])
Hemochromatosis	Ferritin	204 ng/mL (SI: 204 μg/L) (reference range, 12-282 ng/mL [SI: 12-282 μg/L])
	Transferrin saturation	17% (reference range: 12%-45%)
Thyroid	TSH	1.99 μIU/mL (SI: 1.99 mIU/L) (reference range, 0.40-4.40 μIU/mL [SI: 0.40-4.40 mIU/L])
Decreased endogenous insulin	C-peptide	2.96 ng/mL (SI: 0.98 nmol/L) (reference range, 1.12-4.44 ng/mL [SI: 0.37-1.47 nmol/L])
Hyperaldosteronism	Aldosterone/renin	*Not yet resulted

Abbreviations: 5-HIAA, 5-hydroxyindoleacetic acid; C-peptide, connecting peptide; IGF-1, insulin-like growth factor 1; TSH, thyroid stimulating hormone.

## Treatment

Over a 13-month follow-up period, his A1C deteriorated to 9.4% (SI: 79 mmol/mol) despite increasing insulin doses to 400 units of insulin degludec. He did not tolerate doses of semaglutide higher than 1 mg due to gastrointestinal adverse effects, and tirzepatide was unavailable due to widespread pharmacy supply chain shortages. He was evaluated by bariatric surgery but declined to pursue surgery for personal reasons. Due to escalating insulin requirements, the patient was transitioned from insulin degludec to high-potency U-500 insulin at a regimen of 200 units twice daily.

## Outcome and follow-up

After 4 months on U-500 insulin, his A1C improved to 7.7% (SI: 61 mmol/mol) without any other change in pharmacotherapy or lifestyle. While his total daily requirement rose to 400 units of U-500 insulin (the bioequivalent of 2000 units of U-100 insulin), the transition allowed for a substantial reduction in total injection volume. He described being much more satisfied with his glycemic control and reduced injection volumes. Switching from semaglutide to tirzepatide was rediscussed once pharmacy shortages resolved; however, he declined due to a lack of insurance coverage and therefore continued his regimen of semaglutide 1 mg weekly and metformin 1 g twice daily.

## Discussion

Numerous secondary causes of diabetes/insulin resistance exist, which can be classified into 6 broad categories: pancreatic disorders, endocrinopathies, drug- or chemical-induced, genetic, infectious, and immune-mediated [2].

Within pancreatic disorders there is pancreatitis (acute/chronic), prior pancreatic trauma with or without pancreatectomy, neoplasia, hemochromatosis, and severe malnutrition (including pancreatic fibrocalcification) [2]. The pathophysiology of such conditions is mainly an insulin deficiency rather than severe insulin resistance. Screening for such conditions includes careful history and physical in addition to abdominal imaging, lipase and ferritin/iron profile.

Various endocrinopathies can cause secondary insulin resistance. Among these are acromegaly, Cushing syndrome, hyperthyroidism, pheochromocytoma, aldosteronoma, glucagonoma, and somatostatinoma [2]. The pathophysiology behind these endocrinopathies is most commonly hyperglycemia secondary to hormone excess, or in the case of aldosteronoma and somatostatinoma, hypokalemia leading to decreased insulin production [4]. Clinicians can screen for these conditions in suspected cases by ordering an insulin-like growth factor 1 (IGF-1), a dexamethasone suppression test, and/or 24-hour urinary free cortisol, thyroid stimulating hormone (TSH), plasma and urine metanephrines and catecholamines, plasma renin and aldosterone, serum glucagon, and plasma somatostatin, respectively. These tests must be considered in addition to the overall patient presentation. Our patient was known for an adrenal myelolipoma, which raised suspicion for potential mild autonomous cortisol secretion from perhaps a mischaracterized fat-containing adenoma; however, his negative dexamethasone suppression test rendered this possibility less likely.

Medication- or chemical-induced diabetes is another relatively common etiology of secondary insulin resistance. The medications most frequently implicated include glucocorticoids, atypical antipsychotics, calcineurin inhibitors, and highly active antiretroviral therapy [2]. Other commonly prescribed culprit medications include phenytoin, beta-adrenergic agonists, statins, fluoroquinolones, furosemide, thiazides, and thyroid replacement hormone [2].

While rare, there are many possible genetic causes of diabetes. These include defects in beta cell function, such as mitochondrial diabetes (associated with deafness and elevated glycemias), neonatal diabetes [2], and monogenic diabetes, an autosomal dominant phenomenon seen in young patients with diabetes (under 35 years old) who have no/minimal levels of anti-islet and anti-glutamic acid decarboxylase (anti-GAD) antibodies [2]. Genetic mutations causing deficiencies in insulin action are equally possible, such as in insulin receptor defects due to mutation of the *insulin receptor (INSR)* gene which can lead to leprechaunism, Rabson-Mendenhall syndrome, or type A insulin resistance [5]. Finally, many genetic syndromes can predispose to diabetes: Down, Klinefelter, and Turner syndromes being familiar examples [2, 4]. A family history of diabetes and/or obesity is important to elicit to help rule out genetic conditions.

The literature describes numerous infectious causes of diabetes. Invoked viruses include rubella, Coxsackie virus, mumps, cytomegalovirus (CMV) and more common pathogens such as adenovirus [2, 4].

Rare immune-mediated causes are also possible. Stiff-person syndrome is a central neurological autoimmune illness characterized by painful muscle spasms, axial rigidity, and presence of anti-GAD antibodies, though with a different pathophysiology than in type 1 diabetes [6]. Type B resistance is caused by polyclonal immunoglobulin Gs that attack the insulin receptor and result in insulin hypersecretion, usually presenting with the classic triad of high fasting insulin, hypoadiponectinemia, and low-normal triglycerides, and with treatment being centered on immunomodulation [7].

While this case highlights the secondary workup of diabetes, in our patient, no secondary cause was identified. The challenge for clinicians with similar cases then becomes the management to attain glycemic targets.

Our patient was already on >1000 units of insulin per day at the time of referral, as well as metformin and incretin therapy. He had prior intolerance to sodium-glucose co-transporter-2 inhibitors and did not tolerate increasing the dose of his glucagon-like peptide-1 receptor agonist. Despite this regimen and the uptitration of his insulin, his A1C continued to worsen. While bariatric surgery would be indicated at his BMI of 41.7 kg/m^2^ and would assist in the management of severe insulin resistance [8], he had declined this intervention. He was switched to U-500 insulin resulting in a 1.7% (SI: 18 mmol/mol) subsequent decrease in his A1C.

The strongest evidence supporting U-500 insulin comes from 2 randomized controlled trials, the first being one in which 325 patients with type 2 diabetes on >200 units of U-100 were switched to U-500 either twice or thrice daily [9]. This trial showed improvement in A1C of 1.12% to 1.22% at 24 weeks. The VIVID study represents the second trial, in which 420 patients on >200 units of insulin were randomized to U-500 multiple daily injection vs continuous subcutaneous infusion, and showed a decrease in A1C of 0.85% to 1.27% at 24 weeks [10]. A Canadian case series of 17 patients also showed a decrease of 1.6% at 1 year with U-500 [3]. In addition to efficacy, patients themselves appear to prefer U-500 due to the decreased volume of insulin needed [11].

Our case describes the challenges of managing patients with type 2 diabetes and severe insulin resistance, including ruling out secondary causes of insulin resistance and managing pharmacotherapy.

## Learning points

- Glycemic control in the setting of severe insulin resistance can be challenging to manage.
- Concentrated insulin may improve glycemic control and patient satisfaction in the setting of severe insulin resistance.
- Severe insulin resistance should prompt clinicians to investigate for secondary causes of diabetes.

## Contributors

All authors made individual contributions to authorship. B.A. and N.G. were involved in the diagnosis and management of this patient. N.G. obtained informed consent. B.A. drafted the manuscript and N.G. critically reviewed and edited the draft. All authors reviewed and approved the final draft.

## Data Availability

Data sharing is not applicable to this article as no datasets were generated or analyzed during the current study.

## Abbreviations

A1C: glycated hemoglobin
BMI: body mass index
GAD: glutamic acid decarboxylase
